# MaxEnt Modeling and Effects of Climate Change on Shifts in Habitat Suitability for *Sorbus alnifolia* in China

**DOI:** 10.3390/plants14050677

**Published:** 2025-02-22

**Authors:** Yan Luo, Jingshi Yang, Luping Liu, Keliang Zhang

**Affiliations:** College of Horticulture and Landscape Architecture, Yangzhou University, Yangzhou 225009, China; luoyanyzuu@126.com (Y.L.); jingshiyang1@163.com (J.Y.); lupingliu6@163.com (L.L.)

**Keywords:** conservation strategies, environmental factors, future habitat shifts, Rosaceae, species distribution modeling

## Abstract

Anthropogenic climate change stands out as one of the primary forces expected to reshape Earth’s ecosystems and global biodiversity in the coming decades. *Sorbus alnifolia*, which occurs in deciduous forests, is valued for its ornamental appeal and practical uses but is reported to be declining in the wild. Nevertheless, the distribution of this species’ suitable range, along with the key ecological and environmental drivers that shape its habitat suitability, remains largely unknown. By analyzing 198 occurrence records and 54 environmental factors, we employed MaxEnt to project *S. alnifolia*’s current and future habitat suitability. Our results showed that annual precipitation (37.4%), normalized difference vegetation index (30.0%), August water vapor pressure (20.8%), and temperature annual range (3.4%) were the most significant variables explaining *S. alnifolia*’s environmental requirements. The suitable habitats were primarily scattered across eastern and central China. Under projected future climatic conditions, the total expanse of potential habitat is expected to increase. However, most of this expansion involves low-suitability habitats, whereas moderately and highly suitable habitats are likely to shrink, especially in southern and lower-altitude regions of China. Based on these findings, we propose several conservation strategies to support the long-term sustainability of *S. alnifolia*.

## 1. Introduction

Global anthropogenic climate change poses serious risks to biological diversity [1], ecosystem functions [2], and human well-being [3]. According to the report in the IPCC’s Fifth Assessment, global warming is projected to continue for the foreseeable future, with an expected temperature increase of 0.3–4.5 °C by 2100 relative to the 1986–2005 baseline [4]. This ongoing warming, coupled with changes in rainfall patterns and a rise in extreme weather events, is expected to cause mismatches between plants and their optimal growing conditions, affecting their survival, reproductive success, and ability to compete for resources [5,6]. As ecosystems undergo rapid changes, many species face the challenge of adapting to new conditions, while some may experience significant declines or even extinction [7,8]. Consequently, it is essential for forest managers to evaluate how climate variability shapes species’ spatial distributions to identify vulnerabilities and develop effective conservation and management practices [9].

One effective tool for investigating these changes in species distribution is the species distribution model (SDM), which can help predict potential geographic ranges and ecological requirements for species across different climatic contexts [7,10,11,12,13,14,15]. Several SDM techniques, such as bioclimate envelope models [10], Boosted Regression Trees (BRT) [11], CLIMEX [16], ENFA [14], Generalized Linear Models (GLM) [12], Genetic Algorithm for Rule-set Production (GARP) [17,18], MaxEnt [15], and Random Forest (RF) [13] have been used to predict species’ geographic distributions, environmental responses, and ecological requirements. Among these methods, MaxEnt has emerged as a leading and frequently employed method [15,19]. This approach utilizes the principle of maximum entropy to evaluate a species’ presence probability, based on presence-only data and pertinent environmental factors [15]. MaxEnt is highly effective in handling small or incomplete datasets [20,21], accommodating both continuous and categorical variables, and managing complex species–environment interactions [19]. Additionally, MaxEnt provides user-friendly outputs, including probability maps and variable importance metrics, thereby making it invaluable for conservation planning, habitat evaluations, and modeling how climate change may affect biodiversity.

*Sorbus alnifolia* (Siebold & Zuccarini) K. Koch, commonly known as Korean Mountain Ash, is a deciduous tree species in the Rosaceae family, occurring in parts of eastern Asia, including China, the Korean Peninsula, and Japan [22,23]. This species thrives in moist, acidic, and well-drained soils and prefers full sunlight [24,25]. *S. alnifolia* can grow up to 20 m, with a dense, rounded canopy of green leaves that turn vibrant yellow in autumn [22]. In spring, it produces clusters of creamy white flowers, followed by simple, ovate leaves that turn golden-orange hues in the fall. The tree’s striking red-to-orange fruits persist into autumn, providing an important food source for birds [24,25,26]. Additionally, according to the *Flora of China*, the wood of *S. alnifolia* can also be utilized for making vehicles, the bark serves as a dye, and the fibers are used as raw material for paper production [22].

Previous studies on *S. alnifolia* have predominantly focused on wood anatomy [27], pollination biology [26], seed dormancy and germination [23], and its medicinal value [28]. However, there is a lack of comprehensive understanding regarding the species’ habitat preferences, and the environmental factors that influence its distribution remain unclear. Furthermore, recent studies suggest that *S. alnifolia* is experiencing a decline in juveniles or seedlings in field environments, partly due to human disturbances and its deep seed dormancy [23,29]. This decline underscores the need for a thorough understanding of the species’ habitat preferences and the environmental drivers affecting its distribution. Moreover, with ongoing climate change, it is crucial to assess how these shifts may impact *S. alnifolia*’s distribution and sustainability across its native range. Therefore, our study aims to fill these gaps by identifying the key environmental factors shaping its distribution and projecting its future suitability under climate change scenarios.

Using a broad database of geo-referenced *S. alnifolia* observations and 54 environmental variables, encompassing both current and projected climate conditions, we applied MaxEnt modeling across the species’ range in China to provide the first detailed predictions of its spatial distribution and habitat suitability, both current and in the future. The objectives of our study were to (1) identify and quantify the principal environmental factors affecting its distribution; (2) project its potential range under present and future climatic settings (2041–2060 and 2081–2100) by integrating topographic and bioclimatic variables; and (3) estimate and compare the changes in habitat suitability under designated future climate projections, highlighting potential areas of concern and opportunities for conservation efforts. The results obtained by our study are expected to advance our understanding of *S. alnifolia*’s potential range dynamics, offer new insights into climate-induced range shifts that have not been examined before, and contribute valuable information for the development of effective conservation strategies, forest management, and the introduction and cultivation of wild populations under climate change.

## 2. Results

### 2.1. Model Performance

The MaxEnt model achieved an average training AUC of 0.973 ± 0.001 and a Kappa value of 0.926 ± 0.02. These metrics demonstrate the model’s exceptional ability to accurately predict the regional distribution of *S. alnifolia* under current climate conditions in China.

### 2.2. Potential Distribution Regions of Sorbus alnifolia

**Figure 1:**
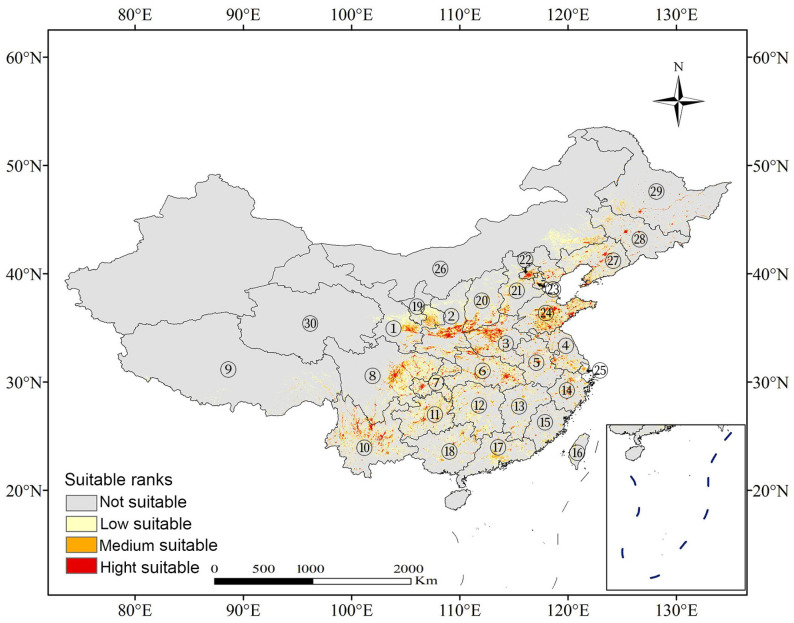
Predicted potential distribution map of *Sorbus alnifolia* under current climate scenarios. ① Gansu; ② Shaanxi; ③ Henan; ④ Jiangsu; ⑤ Anhui; ⑥ Hubei; ⑦ Chongqing; ⑧ Sichuan; ⑨ Xizang; ⑩ Yunnan; ⑪ Guizhou; ⑫ Hunan; ⑬ Jiangxi; ⑭ Zhejiang; ⑮ Fujian; ⑯ Taibei; ⑰ Guangdong; ⑱ Guangxi; ⑲ Ningxia Hui Autonomous Region; ⑳ Shanxi; ㉑ Hebei; ㉒ Beijing; ㉓ Tianjin; ㉔ Shandong; ㉕ Shanghai; ㉖ Inner Mongolia Autonomous Region; ㉗ Liaoning; ㉘ Jilin; ㉙ Heilongjiang; ㉚ Qinghai.

Based on the MaxEnt model, highly suitable habitats (*p* > 0.75) cover approximately 1.05 × 10^5^ km^2^, representing 1.14% of China’s total land area (Table 1; Figure 1). These areas are primarily distributed across eastern and central China, including eastern Hubei, central Shaanxi, Shandong, eastern Sichuan, western Chongqing, western and central Henan, and southern Beijing (Figure 1). Additionally, moderately suitable areas (0.50 ≤ *p* ≤ 0.75) span about 2.38 × 10^5^ km^2^ (2.57% of China’s land), predominantly located in Hubei, Sichuan, Gansu, southern Hunan, central and southern Shandong, northern Henan, and extensive regions of Yunnan. Low-suitability areas (0.25 ≤ *p* < 0.50) account for 8.06 × 10^5^ km^2^ (8.69% of China’s total area) and are distributed across provinces that also contain highly and moderately suitable habitats.

### 2.3. Key Environmental Variables

**Figure 2:**
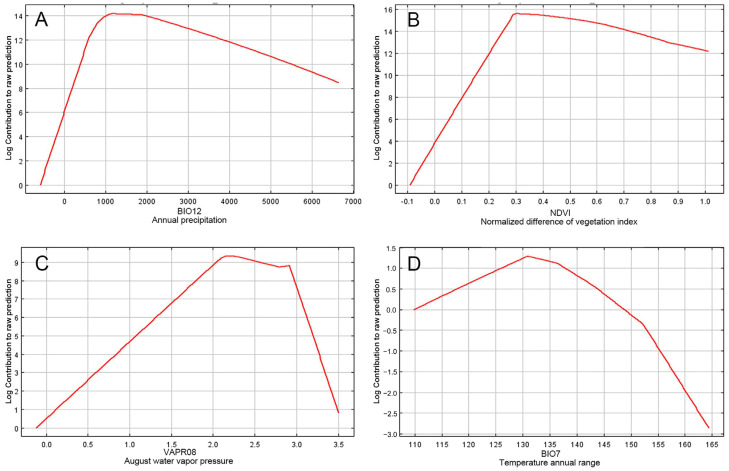
Response curves depicting the effects of key environmental predictors on *Sorbus alnifolia* in the species distribution model. (**A**) Annual precipitation (BIO12, mm); (**B**) normalized difference of vegetation index (NDVI); (**C**) August water vapor pressure (VAPR08, kPa); and (**D**) temperature annual range (BIO7, °C).

The internal jackknife test performed in MaxEnt identified the top four key environmental variables that collectively explained 91.6% of the distributional variance of *S. alnifolia*: BIO12 (37.4%), NDVI (30.0%), VAP8 (20.8%), and BIO7 (3.4%) (Table 2; Figure 2). In contrast, other factors, such as soil bulk density, elevation, slope, and solar radiation contributed only a little to habitat suitability for *S. alnifolia*.

The response curves (with all other variables held constant at their mean values) for these four key variables showed an initial increase in suitability, followed by a decline as each variable approached its optimal range. Specifically, BIO12 showed the highest suitability at precipitation levels between 1000 and 2000 mm (Figure 2A), the NDVI reached its optimal level at around 0.3 (Figure 2B), VAP8 peaked at 2–3.0 kPa (Figure 2C), and BIO7 was most favorable at 13 °C (Figure 2D).

### 2.4. Future Alterations of Suitable Habitat Area

**Figure 3:**
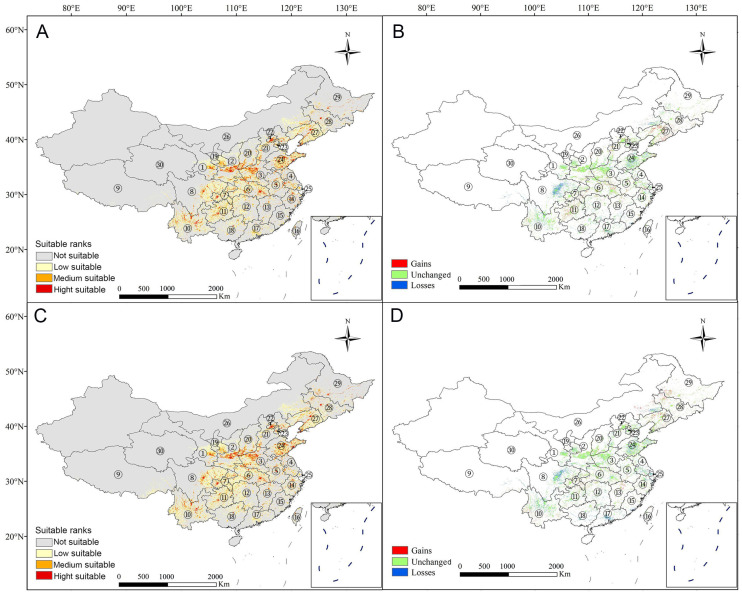
Predicted potential distribution map of *Sorbus alnifolia* under the SSP 245 climate change scenario: the projection for (**A**) 2041–2060 and (**C**) 2081–2100; and a comparison of the current distribution (see Figure 1) with (**B**) the 2041–2060 projection and (**D**) the 2081–2100 projection. ① Gansu; ② Shaanxi; ③ Henan; ④ Jiangsu; ⑤ Anhui; ⑥ Hubei; ⑦ Chongqing; ⑧ Sichuan; ⑨ Xizang; ⑩ Yunnan; ⑪ Guizhou; ⑫ Hunan; ⑬ Jiangxi; ⑭ Zhejiang; ⑮ Fujian; ⑯ Taibei; ⑰ Guangdong; ⑱ Guangxi; ⑲ Ningxia Hui Autonomous Region; ⑳ Shanxi; ㉑ Hebei; ㉒ Beijing; ㉓ Tianjin; ㉔ Shandong; ㉕ Shanghai; ㉖ Inner Mongolia Autonomous Region; ㉗ Liaoning; ㉘ Jilin; ㉙ Heilongjiang; ㉚ Qinghai.

Under the SSP245 climate change scenario, projections indicate that by the 2060s the total suitable habitat for *S. alnifolia* is expected to expand to approximately 13.30 × 10^5^ km^2^ (Figure 3). This expansion includes 9.97 × 10^5^ km^2^ of low suitability, 2.33 × 10^5^ km^2^ of moderate suitability, and 1.00 × 10^5^ km^2^ of high suitability. Compared with current conditions, this represents an increase of 1.81 × 10^5^ km^2^, primarily in Liaoning, Guizhou, southern Jiangsu, northern Hubei, and western Yunnan. By the 2100s, the suitable habitat is expected to reach 13.64 × 10^5^ km^2^, marking a total increase of 2.14 × 10^5^ km^2^ relative to current conditions. Significant expansions are projected in southern Yunnan and Chongqing, central and eastern Liaoning, western Heilongjiang and Jilin, and the southeastern Inner Mongolia Autonomous Region. However, despite the overall increase in suitable habitat, both moderately and highly suitable zones are expected to decline by 0.05 × 10^5^ km^2^ by the 2060s and by 0.16 × 10^5^ km^2^ (moderately suitable areas) and 0.06 × 10^5^ km^2^ (highly suitable areas) by the 2100s. These declines mainly occur in southern China or low-altitude areas, such as southeastern Guangdong and eastern Sichuan (Figure 3).

## 3. Discussion

### 3.1. Prediction of the Current Distribution of Sorbus alnifolia

According to the *Flora of China*, *Sorbus alnifolia* exhibits a broad distribution across numerous provinces in China, including Anhui, Fujian, Gansu, Hebei, Heilongjiang, Henan, Hubei, Hunan, Jiangsu, Jiangxi, Jilin, Liaoning, Shaanxi, Shandong, Shanxi, Sichuan, Taiwan, and Zhejiang [22]. Our MaxEnt modeling results revealed that the combined areas of moderate and high suitability encompass approximately 3.43 × 10^5^ km^2^ under current climatic conditions. This estimation generally aligns with the *Flora of China* [22], indicating the reliability of our predictive model. Land managers and conservationists can use our predicted maps to prioritize areas for reforestation and habitat restoration projects. In addition, our analysis identified certain regions within Yunnan, Guangxi, and the Ningxia Hui Autonomous Region as potentially suitable habitats for *S. alnifolia*. These areas were not previously recognized as primary distribution zones, which could be due to dispersal limitations or past land-use changes that have reduced habitat continuity [26,30,31], suggesting untapped opportunities for introducing the species to these regions.

### 3.2. Environmental Factors Affecting Sorbus alnifolia Distribution

Our results show that the top four environmental variables shaping the habitat suitability of *S. alnifolia* are annual precipitation (BIO12), the normalized difference vegetation index (NDVI), August vapor pressure (VAP8), and the annual temperature range (BIO7). Precipitation and temperature are major determinants of the distribution of many species (e.g., refs. [7,8,30]). Adequate precipitation ensures that water-dependent processes, such as photosynthesis and nutrient uptake, occur efficiently, while optimal temperature ranges regulate metabolic activities and developmental cycles [32,33,34,35]. In our study, BIO12 showed optimal suitability around 1000–2000 mm, which is relatively high compared with other species in eastern China, such as *Paeonia veitchii*, which ranges from 350 to 1200 mm [8]. Such high precipitation supports the fact that *S. alnifolia* is most abundant in wetter climates [22]. Conversely, regions with lower precipitation show low suitability, suggesting that water scarcity limits the establishment and survival of *S. alnifolia* in those areas. Consistently, greenhouse studies also indicate that under drought stress, *S. alnifolia* exhibits reduced leaf area, growth, and photosynthetic capacity [33], and extended dry spells can also impede its recruitment [33]. Similarly, BIO7, although contributing only 3.4% to the overall variation, also plays a crucial role; it influences key life history processes such as seed germination, bud differentiation, and overall plant health [18,23,36]. For example, Tang et al. [23] found that *S. alnifolia* seeds need about 150 days of cold stratification to overcome dormancy, and exposure to very warm temperatures immediately after stratification can actually induce secondary dormancy. Overall, our results indicate that *S. alnifolia* favors climates without pronounced extremes and with ample precipitation, reflecting its physiological tolerances and reproductive ecology.

Moreover, August vapor pressure, an integrative variable reflecting both temperature and precipitation, further delineates the suitable habitats for *S. alnifolia*. Our results show that the probability of occurrence declines sharply when VAP8 drops below 2.0 kPa. In contrast to other species in eastern China, such as *Pistacia chinensis*, which can survive at approximately 1.0 kPa [37], *S. alnifolia* requires higher vapor pressure values to maintain a moist atmosphere and lower vapor pressure deficits during the warm season [38]. This high vapor pressure need has been suggested to reduce the evaporative demand on leaves and help to maintain water balance throughout the growing season [39,40]. Moreover, high vapor pressure allows plant stomata to partially close, thereby minimizing transpiration losses and preventing excessive water tension in the xylem [38,39]. This water-saving strategy is particularly important in late summer, when peak temperatures and drying soils intensify stress, suggesting that *S. alnifolia* may be especially vulnerable to climate regimes with increased summer aridity, even if total rainfall does not drastically decrease.

The NDVI was another significant predictor of *S. alnifolia* distribution. The NDVI is an index of vegetation greenness and productivity [41], and high NDVI values in our study area often correspond to well-forested, undisturbed habitats [42], suggesting that habitat quality and forest cover are important for *S. alnifolia*. In practical terms, areas with lush vegetation (high NDVI) likely indicate mature forest communities or dense understory where *S. alnifolia* finds suitable conditions (e.g., shaded microsites). Moreover, the NDVI serves as an integrative indicator of habitat quality and positively correlates with plant leaf area index, ecosystem net primary productivity, and other key canopy attributes [42,43]. Thus, the strong effect of the NDVI in our model likely reflects that *S. alnifolia* requires habitats with high productivity and structural complexity [43], which provide the necessary resources (light regime, nutrients, and symbiotic partners) for its growth. It may also indicate that *S. alnifolia* prefers less disturbed environments [41]; regions with a low NDVI (e.g., agricultural lands or degraded areas) are unsuitable, possibly due to a lack of tree cover or altered microclimates.

### 3.3. Impact of Climate Change on Sorbus alnifolia Distribution and Related Forest Ecosystems

Increasing global temperatures often push some species to relocate toward higher elevations or latitudes [44,45,46], while others adjust through physiological or phenological shifts [47,48]. In our study, under the SSP245 climate change scenario, which represents an intermediate pathway with greenhouse gas emissions remaining at current levels until 2050, followed by a decline that does not reach net zero by 2100 [49], our models predict that *S. alnifolia* will experience an overall expansion of its range. This suggests that the species may adapt physiologically or phenologically as temperatures rise. However, despite an overall increase in suitable habitat, our models predict a decline in both moderately and highly suitable zones. This indicates that while *S. alnifolia* may find new areas of suitability, existing high-quality habitats could become less favorable. This result is consistent with previous studies (e.g., refs. [8,11]). Such shifts may result from intensified climatic stressors in these regions, including extreme temperature fluctuations and altered precipitation regimes that exceed the optimal thresholds for *S. alnifolia* survival and reproduction. Warmer temperatures and greater moisture availability in newly suitable areas could create optimal conditions for the species to flourish, boosting physiological processes such as photosynthesis, respiration, and transpiration [31].

Furthermore, while our model’s results highlight the direct effects of climate warming, several indirect effects should not be overlooked. Shifts in temperature and moisture regimes can alter species interactions and community dynamics, potentially increasing competitive pressures or facilitating the spread of pests and diseases [8,31]. Milder winters, for example, could enable pest populations to persist longer, placing additional stress on *S. alnifolia* and reducing seedling recruitment [31]. Additionally, upward range shifts may disrupt interactions with specialized pollinators adapted to lower elevations, negatively affecting fruit set and long-term reproductive success [50]. Other indirect impacts, such as more frequent extreme weather events such as droughts, heatwaves, and intense storms, could exacerbate stress on *S. alnifolia* and degrade forest habitats [51]. Furthermore, land-use changes and habitat fragmentation may hinder the species’ ability to migrate or maintain viable populations, further complicating its distribution dynamics [52].

### 3.4. Implications for Conservation Plans

Based on our results and the climate change scenario, we suggest the following conservation strategies. First, habitats that remain suitable across future scenarios could serve as crucial refuges for conservation efforts aimed at *S. alnifolia*. Periodic monitoring of these refugia would enable the early detection of shifts in species performance and inform necessary adjustments to conservation strategies [53]. Second, the expansion of suitable habitats for *S. alnifolia* under the SSP245 climate scenario presents valuable opportunities for conservationists and land managers to implement targeted reforestation and habitat restoration projects in these newly suitable areas. By prioritizing regions with high and moderate suitability, efforts can be optimized to ensure the successful establishment and growth of *S. alnifolia*, thereby enhancing biodiversity and ecosystem functionality [54,55]. Third, maintaining habitat connectivity and protecting existing high-quality habitats are essential strategies to mitigate threats from land-use changes and habitat fragmentation. This ensures that *S. alnifolia* populations can migrate and maintain genetic diversity [56,57]. Fourth, creating genetically diverse seed banks and involving local communities in conservation initiatives may improve the adaptability and long-term sustainability of *S. alnifolia* populations [57]. Fifth, working together with local communities, government bodies, and conservation organizations is essential for protecting newly identified habitats from human-induced pressures such as habitat fragmentation and overharvesting, which could threaten the establishment and long-term survival of the species.

### 3.5. Limitations and Future Research Directions

Species distribution models are increasingly utilized to guide forest management in the face of global change [58,59]. Nevertheless, our study clearly has limitations. First, uncertainties are inevitable in climate projections, and the reliance on a single climate model (BCC-CSM2-MR) and emission pathway (SSP245) may constrain the robustness of our projections, given that different models and scenarios could yield varying outcomes [18]. Therefore, incorporating multiple climate models and diverse emission trajectories in future studies would enhance the reliability of distribution forecasts [60,61]. Second, our occurrence data, sourced from online herbarium databases, may suffer from spatial sampling biases despite the implementation of spatial filtering and bias file layers [20]. These biases could influence the accuracy of habitat suitability predictions, particularly in under-sampled or remote regions [62]. Field-based validation through targeted surveys in newly identified suitable areas would help to verify model predictions and refine future projections. Third, due to limited data availability, our models did not include biotic interaction data (e.g., herbivory, competition, and mutualistic relationships) and used some static variables for future projections, all of which may potentially affect the predicted results [63,64]. Future studies should aim to incorporate dynamic biotic factors and more up-to-date environmental data, including land-use indices, to improve model predictions and provide a more comprehensive understanding of species distribution under climate change.

## 4. Materials and Methods

### 4.1. Occurrence Data of Sorbus alnifolia

**Figure 4:**
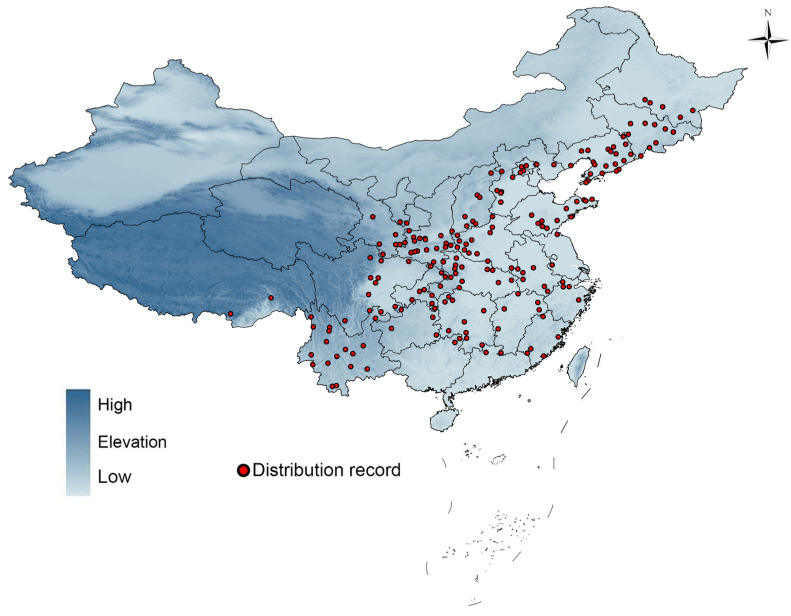
Distribution records of *Sorbus alnifolia* in China.

Occurrence data for *S. alnifolia* were compiled from online herbarium databases, including the Chinese Virtual Herbarium (CVH; [65]), Tropicos [66], and GBIF [67]. These sources are widely recognized for their comprehensive and up-to-date plant occurrence (and/or specimen) data. Nevertheless, some biases may exist due to geographical clustering in regions with higher research efforts and easier access. We hence excluded records without a specified collection site, and for specimens that only provided a location name we determined the longitude and latitude using Google Maps. For all occurrence data, we ensured the accuracy of the locations by verifying their coordinates via Google Maps. During this process, we excluded occurrences from cultivated areas and kept only those from natural habitats. Although we cannot completely rule out the possibility that some occurrences in forested areas are cultivated, this approach helped to minimize the impact of human cultivation (such as irrigation) on the reliability of the subsequent model results. After eliminating duplicate collection sites, we applied spatial filtering to the remaining points to retain only one occurrence within each 1.0 × 1.0 km grid cell. This resolution was chosen to match the 30 arc-seconds climate data resolution (below), ensuring consistency between the occurrence data and environmental variables. This filtering process resulted in 198 occurrence points for modeling the distribution of *S. alnifolia* (Figure 4).

### 4.2. Environmental Variables Used for Model Simulation

A total of 54 environmental variables that could influence the distribution of *S. alnifolia* were used in the modeling (See Appendix A Table A1 for the list of all 54 environmental factors). These variables included 19 bioclimatic factors, which are commonly used in species distribution modeling as they capture key climate influences on species distribution (e.g., refs. [31,36]). Solar radiation and water vapor pressure were also included, with monthly measurements of these factors from the World Climate Database [68]. Solar radiation plays a significant role in photosynthesis, influencing plant energy balance, leaf development, phenology, and overall productivity [69]. High solar radiation levels, especially during the growing season, are crucial for driving photosynthetic activity and energy production in plants, which directly impact their growth and reproductive success [69]. Water vapor pressure, on the other hand, affects plant transpiration and water availability, which are critical for maintaining cellular functions, cooling, and overall growth [39,40]. Both factors are key drivers of plant health and phenology, particularly in relation to climatic conditions [39,69]. Furthermore, three topographic variables (slope, aspect, and elevation) were obtained from RESDC [70] for the period 1984–1995, as topography plays a significant role in determining microclimates and habitat suitability for many species and is unlikely to change significantly in the short term. These factors influence moisture availability, temperature variation, and sunlight exposure, all of which directly affect plant growth [71]. Seven soil properties, including soil organic carbon, pH, bulk density, conductivity, and the percentages of sand, silt, and clay, were obtained from the Harmonized World Soil Database v1.2 [72], as soil characteristics strongly influence plant growth by affecting water retention, nutrient availability, and root penetration. Soil pH, for example, determines nutrient availability, while organic carbon is essential for soil fertility and microbial activity. These soil parameters are critical in shaping the habitat conditions for *S. alnifolia* and other plant species [72]. Finally, NDVI data were sourced from the China Meteorological Data Sharing Service System, as vegetation indices are important for assessing habitat quality, vegetation cover, and the overall health of ecosystems [42,43]. Overall, all of these variables were selected based on their known ecological relevance and their capacity to capture key factors influencing the species’ distribution.

For future projections, climate data from the BCC-CSM2-MR model (CMIP6) under SSP 245 (IPCC) were used to represent conditions for the periods 2041–2060 and 2081–2100 (hereafter referred to as the 2060s and 2080s, respectively). Compared with previous models such as CMIP5, the CMIP6 models more accurately reflect greenhouse gas (GHG) concentrations and offer improved temperature simulations, particularly regarding the atmosphere [73,74]. The BCC-CSM model is recommended for short-term climate forecasting and climate change assessments in China [18,75]. SSP 245 was selected because it represents a moderate emissions scenario that aligns with current global policy trends, assuming a radiative forcing of +4.5 W/m^2^ by 2100 relative to pre-industrial levels [74]. This scenario reflects a balance between ongoing emissions and mitigation efforts, providing a plausible projection for future climate conditions [76]. Other factors used in predicting current distributions, such as soil variables, can change over relatively short timescales due to the impacts of climate change [31]. For example, at a given site, if soil variables change significantly in the future beyond a species’ tolerance, the species may no longer survive there. However, because static variables were used, MaxEnt cannot capture these dynamic changes, which could result in those sites being predicted as suitable for species distribution. As a result, it is crucial to include these variables in future prediction models. However, reliable projections of how these variables will evolve under future climate scenarios are often unavailable. Previous studies have suggested that when dynamic variables are lacking for future predictions, incorporating static variables can yield better results than excluding them [77]. Therefore, we have included these current variables in the modeling for future projections.

To maintain uniformity across all layers, the environmental raster datasets were processed with a consistent spatial extent and the WGS84 projection in ArcGIS 10.0. The raster files were then reprojected onto an equal-area grid with a spatial resolution of 1.0 km. PCA and Pearson’s correlation were conducted to reduce overfitting (see [78] for details). For each pair of highly correlated variables (|r| > 0.85), only one variable was selected for further analysis. The decision on which variable to retain was based on the contribution of each variable to PC1 in the PCA, as this principal component explained more variance than any other principal component [78]. The final set of environmental variables included solar radiation for June, July, September, and October (SRAD6, SRAD7, SRAD9, SRAD10), aspect, BIO2, BIO7, BIO12, August water vapor pressure (VAP8), NDVI, elevation, slope, soil bulk density, soil sand percentage, and soil total organic carbon (Table 2).

### 4.3. Model Simulation and Validation

We employed MaxEnt version 3.3.3k [58] to model the distribution of *S. alnifolia* by integrating species occurrence data with environmental variables. Seventy-five percent of the occurrence points were designated for model training, while the remaining 25% were allocated for validation.

Sampling bias in MaxEnt can lead to overfitting, as the model may emphasize areas with overrepresented environmental conditions, potentially skewing predictions and reducing its ability to generalize to unobserved areas with different environmental conditions [15,19]. To address this issue, we generated a bias file layer using a Gaussian kernel density surface implemented via the ‘kde2d’ function in R (v4.4.1). This function employs a bivariate normal kernel with a diagonal bandwidth matrix, where the bandwidth parameters are optimally chosen without assuming any parametric model for the data. Bandwidth values were determined using Silverman’s rule of thumb, which assumes that the underlying density follows a normal (Gaussian) distribution to capture the spatial scale of the local sampling effort [79]. This bias file, scaled from 1 to 20, was then used in MaxEnt to weight background sampling by assigning higher probabilities to areas with denser occurrence records, thereby reducing the overrepresentation of well-sampled regions and yielding distribution maps that more accurately reflect true habitat suitability [15,77].

Previous studies have indicated that the default settings of MaxEnt might not be optimal, particularly when occurrence data are limited [20], and values < 1 may lead to overfitting, while larger values may reduce the model’s ability to capture environmental variation. However, after testing various regularization multiplier values (i.e., 1–5), we found that the default setting (i.e., 1) provided the best performance, accurately representing the known distributions of *S. alnifolia* without leading to overfitting. We limited the number of background points to 10,000, as increasing this number to 100,000 did not enhance model performance. Additionally, we activated the “fade by clamping” feature in MaxEnt to address areas affected by clamping. The maximum number of iterations was set to 1000 to ensure model convergence, with a convergence threshold of 1 × 10⁻^6^ [31]. We retained the default “autofeatures” option, which includes linear, quadratic, hinge, threshold, and product features [20].

Given that our dataset was compiled from multiple sources and may contain some unknown inaccuracies, we adopted the 10th percentile training presence logistic threshold (as suggested by refs. [58,80]) to define the minimum probability of suitable habitat for *S. alnifolia*. This threshold excludes the lowest 10% of occurrence records, retaining the top 90% of reliable data, and provides a more balanced and ecologically meaningful estimate compared with alternative thresholds, such as the minimum training presence or other fixed threshold methods [58]. We utilized response curves to interpret the MaxEnt outputs and evaluated the significance of each environmental variable through jackknife tests. Model performance was assessed using both the AUC and the Kappa statistic. The AUC was calculated for both training and testing datasets, where values range from 0 to 1. An AUC of 0.5 indicates performance no better than random, while values approaching 1 reflect excellent model performance. Models with AUC values above 0.7 were considered acceptable [81]. The AUC is a widely used metric for evaluating the overall accuracy of a model, particularly its ability to distinguish between suitable and unsuitable habitats. In addition to the AUC, we also calculated the Kappa statistic, a threshold-independent metric that ranges from −1 to 1. A value of 0 indicates performance no better than random, while higher values reflect better model performance [82]. The Kappa statistic is often used to assess agreement between predicted and observed data, offering an alternative measure of accuracy, especially when the dataset contains imbalanced classes. Both metrics were chosen to provide a comprehensive evaluation of model performance, ensuring reliability and robustness in our results.

Habitat suitability was classified into four categories: high (>0.75), moderate (0.50–0.75), low (0.25–0.50), and unsuitable (<0.25), based on probability values between 0 and 1. After modeling the current suitable habitat for *S. alnifolia* using present climatic data, we projected suitable habitat extents under future climate scenarios. We compared the projected future habitat areas for *S. alnifolia* with its current distribution and identified regions that had become unsuitable, become suitable, or remained unchanged.

## 5. Conclusions

Accurate distribution data are essential for prioritizing conservation efforts and implementing strategies to effectively address climate change. Using MaxEnt modeling, we evaluated *S. alnifolia*’s present and future distribution in China under the SSP245 climate change scenario. Our findings indicate that the key environmental factors shaping the distribution of *S. alnifolia* include annual precipitation (37.4%), normalized difference vegetation index (30.0%), August water vapor pressure (20.8%), and temperature annual range (3.4%). At present, moderately and highly suitable habitats span approximately 3.43 × 10^5^ km^2^, concentrated in eastern and central China. In the future, our findings suggest a significant expansion of suitable habitats by the 2060s and 2100s. Nevertheless, despite this overall growth, moderately and highly suitable areas are expected to decline, particularly in southern China and low-altitude regions.

## Figures and Tables

**Table 1:** Proportions of the three habitat suitability categories for the potential distribution of *Sorbus alnifolia* under current and SSP245 climate scenarios.

	Area (×10^5^ km^2^)			Portion of Area (%)		
	Low Suitable	Moderate Suitable	High Suitable	Low Suitable	Moderate Suitable	High Suitable
Current	8.06	2.38	1.05	8.69	2.57	1.14
2060s	9.97	2.33	1.00	10.76	2.51	1.08
2100s	10.43	2.22	0.99	11.25	2.39	1.07

**Table 2:** Percentage contributions and permutation importance of the environmental variables incorporated into the MaxEnt models for *Sorbus alnifolia*.

Variable	Bioclimatic Variables	Contribution (%)	Permutation Importance
BIO12	Annual precipitation	37.4	50.6
NDVI	Normalized difference of vegetation index	30	22
VAP8	Water vapor pressure of August	20.8	16.7
BIO7	Temperature annual range	3.4	2.4
SLOP	Slope degree	2.6	4
SRAD7	Solar radiation of July	1.8	1.1
ELEV	Elevation	1.1	0.6
ASP	Aspect	0.7	0.9
SRAD6	Solar radiation of June	0.5	0.4
SAND	Soil sand percentage	0.4	0.6
BIO2	Mean diurnal range	0.4	0.4
SRAD10	Solar radiation of October	0.3	0.1
BUCK	Soil buck density	0.2	0.1
SRAD9	Solar radiation of September	0.2	0
TOC	Soil total organic carbon	0.1	0.1

## Data Availability

Data are contained within the article.

## Appendix A

**Table A1:** Environmental variables used in the species distribution modeling of *Sorbus alnifolia*.

Category	Variable Name	Unit	Abbreviation	Source
Bioclimatic	Annual Mean Temperature	°C	BIO1	WorldClim 2
	Mean Diurnal Range (Mean of monthly (max temp–min temp))	°C	BIO2	WorldClim 2
	Isothermality (BIO2/BIO7) (×100)	-	BIO3	WorldClim 2
	Temperature Seasonality (standard deviation ×100)	°C	BIO4	WorldClim 2
	Max Temperature of Warmest Month	°C	BIO5	WorldClim 2
	Min Temperature of Coldest Month	°C	BIO6	WorldClim 2
	Temperature Annual Range (BIO5-BIO6)	°C	BIO7	WorldClim 2
	Mean Temperature of Wettest Quarter	°C	BIO8	WorldClim 2
	Mean Temperature of Driest Quarter	°C	BIO9	WorldClim 2
	Mean Temperature of Warmest Quarter	°C	BIO10	WorldClim 2
	Mean Temperature of Coldest Quarter	°C	BIO11	WorldClim 2
	Annual Precipitation	mm	BIO12	WorldClim 2
	Precipitation of Wettest Month	mm	BIO13	WorldClim 2
	Precipitation of Driest Month	mm	BIO14	WorldClim 2
	Precipitation Seasonality (Coefficient of Variation)	-	BIO15	WorldClim 2
	Precipitation of Wettest Quarter	mm	BIO16	WorldClim 2
	Precipitation of Driest Quarter	mm	BIO17	WorldClim 2
	Precipitation of Warmest Quarter	mm	BIO18	WorldClim 2
	Precipitation of Coldest Quarter	mm	BIO19	WorldClim 2
Solar Radiation	Solar Radiation January	kJ/m^2^/day	SRAD1	WorldClim 2
	Solar Radiation February	kJ/m^2^/day	SRAD2	WorldClim 2
	Solar Radiation March	kJ/m^2^/day	SRAD3	WorldClim 2
	Solar Radiation April	kJ/m^2^/day	SRAD4	WorldClim 2
	Solar Radiation May	kJ/m^2^/day	SRAD5	WorldClim 2
	Solar Radiation June	kJ/m^2^/day	SRAD6	WorldClim 2
	Solar Radiation July	kJ/m^2^/day	SRAD7	WorldClim 2
	Solar Radiation August	kJ/m^2^/day	SRAD8	WorldClim 2
	Solar Radiation September	kJ/m^2^/day	SRAD9	WorldClim 2
	Solar Radiation October	kJ/m^2^/day	SRAD10	WorldClim 2
	Solar Radiation November	kJ/m^2^/day	SRAD11	WorldClim 2
	Solar Radiation December	kJ/m^2^/day	SRAD12	WorldClim 2
Water Vapor Pressure	Water Vapor Pressure January	kPa	VAP1	WorldClim 2
	Water Vapor Pressure February	kPa	VAP2	WorldClim 2
	Water Vapor Pressure March	kPa	VAP3	WorldClim 2
	Water Vapor Pressure April	kPa	VAP4	WorldClim 2
	Water Vapor Pressure May	kPa	VAP5	WorldClim 2
	Water Vapor Pressure June	kPa	VAP6	WorldClim 2
	Water Vapor Pressure July	kPa	VAP7	WorldClim 2
	Water Vapor Pressure August	kPa	VAP8	WorldClim 2
	Water Vapor Pressure September	kPa	VAP9	WorldClim 2
	Water Vapor Pressure October	kPa	VAP10	WorldClim 2
	Water Vapor Pressure November	kPa	VAP11	WorldClim 2
	Water Vapor Pressure December	kPa	VAP12	WorldClim 2
Topographic	Slope	degrees	SLOP	RESDC
	Aspect	degrees	ASP	RESDC
	Elevation	meters	ELEV	RESDC
Soil	Soil Organic Carbon	g/kg	TOC	Harmonized World Soil Database v1.2
	Soil pH	-	pH	Harmonized World Soil Database v1.2
	Bulk Density	g/cm^3^	BUCK	Harmonized World Soil Database v1.2
	Electrical Conductivity	dS/m	EC	Harmonized World Soil Database v1.2
	Sand Percentage	%	Sand	Harmonized World Soil Database v1.2
	Silt Percentage	%	Silt	Harmonized World Soil Database v1.2
	Clay Percentage	%	Clay	Harmonized World Soil Database v1.2
Vegetation	Normalized Difference Vegetation Index	-	NDVI	China Meteorological Data Sharing Service System
